# *Helicobacter pylori* (*H. pylori*) Infection-Associated Dyslipidemia in the Asir Region of Saudi Arabia

**DOI:** 10.3390/life13112206

**Published:** 2023-11-13

**Authors:** Mohammad Asrar Izhari, Omar A. Al Mutawa, Ali Mahzari, Essa Ajmi Alotaibi, Maher A. Almashary, Jaber Abdullah Alshahrani, Ahmed R. A. Gosady, Abdulrahman M Almutairi, Daifallah M. M. Dardari, Abdul Kareem A. AlGarni

**Affiliations:** 1 Department of Laboratory Medicine, Faculty of Applied Medical Sciences, Al-Baha University, Al Baha 65522, Saudi Arabia; 2 Department of Dermatology, Al-Kharj Military Hospital, Al-Kharj 11361, Saudi Arabia; 3 Medical Services Department, Armed Forces Medical Services, Riyadh 11159, Saudi Arabia; 4 Department of Medical Training and Education, Armed Forces Hospital Southern Region, Khamis Mushait 62413, Saudi Arabia; 5 Laboratory Department, Baish Primary Healthcare, Jazan 87311, Saudi Arabia; 6 Health facilities Infection Control Department, General Directorate of Health, Al-Baha 11134, Saudi Arabia; 7 Laboratory Department, Baish General Hospital, Jazan 87732, Saudi Arabia; 8 Department of Hematology, King Saud Bin Abdulaziz University for Health Sciences, Al-Ahsa 11451, Saudi Arabia

**Keywords:** dyslipidaemia, infection, *H. pylori*, association, hypercholesterolemia, logistic regression

## Abstract

Objectives: *H. pylori*-associated dyslipidemia has been reported to be a major risk factor for atherosclerosis and coronary heart diseases. We aimed to investigate the association of the *H. pylori* infection with dyslipidemia. Methods: A retrospective case–control study was undertaken to evaluate *H. pylori*-associated dyslipidemia, where *H. pylori*-positive individuals were treated as the case group (*n* = 260) while *H. pylori*-negative individuals were considered as the control group (*n* = 250). The mean ± SD of the age of the patients included (*n* = 510) was 44.01 ± 13.58 years. Study subjects with a total cholesterol level of >5.17 mmol/L and/or a triglyceride level of >1.69 mmol/L and/or an LDL-C level of >2.59 mmol/L and/or an HDL-C level of <1 mmol/L in males and/or an HDL-C level of <1.3 mmol/L in females were defined as dyslipidemia. Descriptive (mean, standard deviation, median, and IQR) and inferential (*t*-test, chi-square test, and logistic regression) statistical analyses were undertaken using the R-base/R-studio (v-4.0.2)/tidyverse package. Univariate and bivariate logistic regressions were executed to calculate the crude and adjusted odds ratio along with the *p*-value. A *p*-value of <0.05 was the cut-off for statistical significance. We used ggplot2 for data visualization. Results: The differences in overall mean ± SD (*H. pylori* positive vs. negative) of the cholesterol (5.22 ± 1.0 vs. 5.49 ± 0.85, *p* < 0.01), triglyceride (1.66 ± 0.75 vs. 1.29 ± 0.71, *p* < 0.001), LDL-C (3.43 ± 0.74 vs. 3.26 ± 0.81, *p* < 0.05), and HDL-C (1.15 ± 0.30 vs. 1.30 ± 0.25, *p* < 0.001) levels were statistically significant. The cholesterol and LDL-C levels in ages >60, age = 30–60, in females, and LDL-C levels in males were not significantly different for the *H. pylori*-positive and -negative groups. The proportion (*H. pylori* positive vs. negative) of hypercholesterolemia (190/59.9% vs. 127/40% *p* < 0.01), hypertriglyceridemia (136/68% vs. 64/32% *p* < 0.001), high LDL-cholesterolemia levels (234/53% vs. 201/46% *p* < 0.01), and low HDL-cholesterolemia levels (149/71% vs. 60/28.7% *p* < 0.01) were statistically significant. The odds of having hypercholesterolemia (AOR: 2.64, 95%CI: 1.824–3.848, *p* < 0.001), hypertriglyceridemia (AOR: 3.24, 95%CI: 2.227–4.757, *p* < 0.001), an increased LDL-C level (AOR: 2.174, 95%CI: 1.309–3.684, *p* < 0.01), and a decreased HDL-C level (AOR: 4.2, 95%CI: 2.937–6.321, *p* < 0.001) were 2.64, 3.24, 2.17, and 4.2 times higher in the *H. pylori*-infected individuals as compared with the *H. pylori*-uninfected group. Conclusion: Our results demonstrate that an enhanced risk of dyslipidemia is associated with the *H. pylori* infection, which can aggrandize the atherosclerosis process. The evaluation of temporal variation in the lipid profile in *H. pylori*-infected individuals is recommended for the effective management of *H. pylori*-infected patients.

## 1. Introduction

*H. pylori* (spiral-shaped, microaerophilic Gram-negative bacteria) [1] causes widespread infection in almost half (50%) of the global population [2]. Although the prevalence of *H. pylori* infection is ubiquitous, high geographic variability has been reported [3]. The prevalence of *H. pylori* infection among adults in different geographical regions of Saudi Arabia ranges from 28–46% while 40% has been documented in children [4]. Moreover, the highest prevalence has been reported in the southern region of Saudi Arabia [5]. *H. pylori* infection is in a hyper-endemic state in Saudi Arabia and it affects all age groups [6]. In the majority of the cases, the individual remains asymptomatic for a longer period, fostering a prolonged colonization and debilitation of gastric mucosa [7] that triggers the development of various diseases in the gastrointestinal tract [8]. 

Predominantly, *H. pylori* causes chronic gastritis, peptic ulcers [9], gastric carcinogenesis [10], as it is considered a class 1 carcinogen, and antrum gastritis [11]. In addition to this, the International Agency for Research on Cancer (IARC) has identified *H. pylori* as a class 1 carcinogen [12]. In recent years, the implication of *H. pylori* infection in extra-gastric clinical manifestations such as IDA [13], immune thrombocytopenia [14] dyslipidemia [15], atherosclerosis, cardiovascular disease, neurological disorders, dermatological diseases, and ophthalmic and metabolic diseases [2] have been reported. 

An avalanche of epidemiological studies and clinical reports has delineated the significant association of *H. pylori* with risk factors for cardiovascular diseases [16,17]. A very recently published observational cohort meta-analysis report by Sun et al. has demonstrated the increased risk of developing CVD in *H. pylori*-infected patients [18]. However, the association was not confirmed in various epidemiological investigations [19,20], and the association of *H. pylori* with the risk of enhanced cardiovascular disease is not fully understood and remains controversial [18]. The human microbiome plays a key role in lipid metabolism and is closely related to dyslipidemia [21]. Plasma lipid and lipoprotein abnormality constitute dyslipidemia and are metabolically interrelated [22]. The *H. pylori*-triggered inflammatory response alters the nutrient absorption and serum lipid profile [23]. The intestinal absorption of these factors is crucial to maintain their optimum level in the body [24]. Low-density lipoprotein cholesterol (LDL-C), high-density lipoprotein (HDL), triglycerides, and cholesterol together constitute the serum lipid profile [25]. Dyslipidemia is one of the well-known risk factors for coronary heart disease and heart stroke [26]. An elevation in any of the lipid profiles, for instance triglycerides (TGs), total cholesterol (TC), and low density lipoprotein cholesterol (LDL-C) or a drop in the level of high density lipoprotein cholesterol (HDL-C), is termed as dyslipidemia (hyper-dyslipidemia and hypo-dyslipidemia) [27] which may contribute to atherosclerosis and cardiovascular diseases (CVDs) [2,28]. Dyslipidemia has been reported to be one of the most crucial risk factors for CVDs [29]. Atherogenic dyslipidemia is an important risk factor for myocardial infarction which may lead to a stroke [30]. 

The determination of the lipid profile is necessary for the diagnosis and management of dyslipidemia, atherosclerosis risk, and cardiovascular diseases [31]. Hypercholesterolemia brings about 33% of the global ischemic heart diseases that lead to an estimated 2.6 million deaths worldwide [15]. The implication of *H. pylori* infection in the alteration of the serum lipid profile has been reported. Gastric colonization and invasion by *H. pylori* trigger disturbances in the lipid profile as a result of impaired coagulation cascade activation, autoimmunity due to antigenic molecular mimicry between human epitopes and that of *H. pylori*, the impairment of nutritional absorption, and an inflammatory response to the infection [32,33]. A thorough investigation of the association of *H. pylori*-associated dyslipidemia is worthy of being addressed to lay down the policy for the effective management of *H. pylori*-infected patients. Therefore, the current study aimed to assess the association of *H. pylori* infections with dyslipidemia in a population of the Asir region of the Kingdom of Saudi Arabia.

## 2. Methods and Materials

### 2.1. Data acquisition, Study Area, Design, Population, and Period

An observational study design (hospital-dependent comparative case-control study) was adopted in the current study to evaluate the extra-gastric impact of *H. pylori* in a population of the Asir region of the Kingdom of Saudi Arabia. Laboratory investigation reports of *H. pylori*-infected patients and *H. pylori*-negative cases available at Armed Forces Hospitals of the southern (Asir) region of Saudi Arabia for approximately six years (1 June 2017 to 27 March 2023) were thoroughly examined, reviewed, and screened for demographic, CBC, lipid profile, ferritin, and other metabolic parameter data. Relevant data were plugged into MS Office 365 (an Excel sheet) for cleaning, stratification, and processing. Medical records of all the hospitals of the Asir region, namely, the King Fahad Military Hospital (Khamis Mushait), King Faisal Military Hospital (Military City, Khamis Mushait), Family & Community Medical Center Asir Base, (Khamis Mushait), Family & Community Medical Center Aba (Abha City), and Family & Community Medical Center Ahad Rafidah, (Ahad Rafidah province) were included in this study for data mining.

### 2.2. Eligibility (Inclusion) Criteria

*H. pylori*-infected patients and the *H. pylori*-negative cases of males, females, and all age groups were included in this study. 

### 2.3. Eligibility (Exclusion) Criteria

For excluding participants from this study, historical background, clinical condition, therapeutic, and laboratory records present in the hospitals were examined critically. Based on body mass index (BMI), the A hemoglobin A1C (HbA1C) cut off laid down by the ministry of health, Saudi Arabia for obesity and diabetes (HbA1C ≥ 6.5%) patients were excluded. Following the examination of therapeutic records of the hospital patients on lipid-lowering medications, antiviral treatment was excluded. Participants with tuberculosis, hypertension (blood pressure exceeding 130/80 mm of Hg), kidney transplants, and pregnant women as well as those severely sick with multiple comorbidities (Hemodialysis and intensive care unit patients: admitted patients with obesity, hepatopathy, nephropathy, and *H. pylori*-infected gastric cancer patients that were kept on the prolonged use of a proton pump inhibitor as well as patients on corticosteroid therapy) were excluded by inquisitively examining their clinical records to maintain the homogeneity of the participants and the internal validity and generalizability of the findings of this study. The use of corticosteroid, proton pump inhibitors and other medicines was assessed by a robust system of the drug and therapeutic committee (DTC), in collaboration with pharmacists and physicians at the hospital to ensure drug safety and rational drug use based on the patients’ physiological conditions. Corticosteroid and PPIs are lipid-interfering drugs, and their prolonged use could be one of the confounding factors in this study; therefore, such participants were excluded from this study, and the assessment of prolonged use was accomplished by DTC. 

### 2.4. Determination of Sample Size

**Figure 1:**
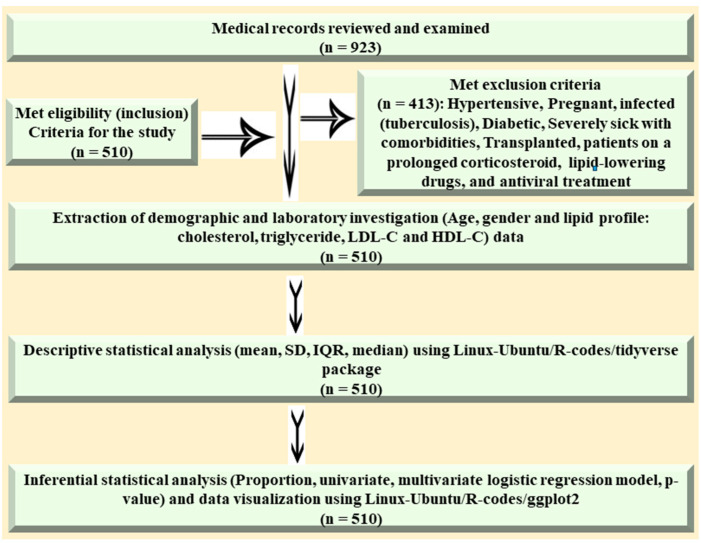
Illustration of a concept map of the method: sample size, study parameters, analysis techniques, and data visualization resources.

The sample size for this study was determined by applying the standard statistical equation *n* = N/(1 + Ne^2^). A 95% confidence level and a margin of error of 0.05 was taken into consideration for the determination of sample size [34]. A sample size of approximately *n* = 400 was determined appropriate for this study based on the standard statistical equation *n* = N/(1 + Ne^2^) used. A total of *n* = 923 hospital records were reviewed and examined thoroughly, and *n* = 413 were excluded from this study as they did not meet the inclusion criteria. In the current study, a sample size of *n* = 510 was taken into consideration to compensate for the sampling variability (Figure 1). 

### 2.5. Operational/Case Definition

Dyslipidemia was characterized when the total cholesterol level was >5.17 mmol/L and/or the triglyceride level was >1.69 mmol/L and/or the LDL-C level was >2.59 mmol/L and/or the HDL-C level was <1 mmol/L in males and/or the HDL-C level was <1.3 mmol/L in females [23,35]. 

### 2.6. Data Generation and Laboratory Techniques

Demographic data (gender and age in years) of all the study participants were obtained from the medical records of all the hospitals included in this study. Primarily, fasting blood specimens and stool specimens were collected at the respective laboratory collection centers of all the hospitals included in this study by applying standard collection procedures, as per the standard operating procedures (SOPs) of the hospitals. The stool specimens were collected aseptically in sterile collection containers. Blood was further processed to separate serum by centrifugation for analysis. Liver function tests (LFTs) and the lipid profile, HDL-C, LDL-C, TG, and TC, were measured by the D×C 700 AU auto-analyzer (Beckman Coulter, Brea, CA, USA) by using commercially available kits [36]. AU is a recent variant with combined hardware- and software-integrated characteristics. Software-based validation is automated by the execution of an analytical measurement range (AMR) and/or linearity along with intra-run and inter-run precision/accuracy checks, the verification of sensitivity (low detection limit), carryover acceptability, and a reference range [36]. All the test specimens were processed as per the manufacturer’s instructions and laboratory standard procedures. Before processing the test samples, the analyzer had to pass the system setup, calibration module, and quality control (QC) testing with positive and negative controls to obtain the validated results of the parameters assessed. Meanwhile, for measuring the complete blood count (CBC), the ADVIA 2120i hematology system was used. Ferritin was determined by applying the D × I 800 auto-analyzer (Beckman Coulter, Brea, CA, USA). Quality control was maintained as per the manufacturer’s instructions prior to testing each specimen. 

The exposure was ascertained by using the sole method of diagnosis of *H. pylori* infection based on the detection of *H. pylori* antigens in the stool samples of the participants, which was termed as the stool antigen tests (SATs). SATs are non-invasive modules for the diagnosis of *H. pylori* infection, as per both European and Japanese guidelines [37]. For the detection of the antigen *H. pylori* in stool specimens, the rapid immune-chromatographic test was undertaken using the Immuno CARD STAT (Meridian Bioscience. Inc., Cincinnati, OH, USA), HPSA ztest procedure. The stool specimens were diluted with a sample diluent and applied to the test device. The loaded test device was incubated at 20–26 degrees Celsius for five minutes. The results of the samples along with those of the positive and negative controls were recorded and interpreted. The test card contains in-built positive and negative controls to validate the results of antigen detection in stool samples for the primary diagnosis of H. pylori infection [37].

### 2.7. Data Quality Assurance and Management

The data collector/investigator was trained to identify and remove any technical biases. Multiple reviews were carried out to ensure the completeness of the demographic and laboratory data. Discrepant and/or incomplete data were eliminated from this study. 

### 2.8. Statistical Analysis of Data and Interpretation

Data were stratified by gender and age categories (age < 30 years, age = 30–60 years, and age > 60 years) before the data analysis. *H. pylori* antigen-positive participants were considered as a case while antigen-negative participants were treated as a control group. Descriptive data analysis was undertaken by applying R-base/R-studio (v-4.0.2) and an Ubuntu/Linux-based machine to measure the mean, median, and interquartile range (IQR) of all the stratified groups in the case of continuous variables, which were summarized in a table. Results retrieved from the analysis of categorical variables were summarized as frequency and proportion/percentage. Using the R-base/R-studio (v-4.0.2)/tidyverse package, inferential statistical analyses *(t*-test, chi-square test, univariate, and multivariate logistic regressions) were accomplished. *p*-values of <0.05 were considered as cut-off values for statistical significance. For data visualization, we used different packages of R, such as ggplot2. 

## 3. Results 

### 3.1. Baseline Characteristics (Demographic and Lipid Profile) of the Study Participants

The data retrieved for all the participants (*n* = 510) were stratified into six categories (two categories by gender, three categories by age, and one overall category). Following stratification, descriptive statistical analyses were accomplished on the demographic, lipid profile (cholesterol, triglyceride, LDL-C, and HDL-C), liver function test (LFT), and HbA1c data for all the six categories to determine the mean, median, standard deviation (SD), and interquartile range to represent the shape and characteristics of the overall data included in this study, which have been tabulated systematically in Table 1. The mean ± SD age for the overall, male, and female categories were 44.01 ± 13.58, 43.31 ± 13.81, and 44.69 ± 13.33, respectively. The mean ± SD cholesterol levels of the overall, male, and female categories and for ages < 30, age = 30–60, and ages > 60 were found to be 5.36 ± 0.96, 5.38 ± 1.02, 5.34 ± 0.92, 4.78 ± 0.89, 5.47 ± 0.93, and 5.38 ± 1.05, respectively. The mean ± SD triglyceride levels of the overall, male, and female categories and for ages < 30, age = 30–60, and ages > 60 were considered to be 1.49 ± 0.72, 1.60 ± 0.74, 1.37 ± 0.68, 1.18 ± 0.65, 1.52 ± 0.73, and 1.20 ± 0.66, correspondingly. The mean ± SD HDL-C and LDL-C levels for the overall, male, and female categories and for ages < 30, age = 30–60, and ages > 60 were 1.23 ± 0.29, 1.11 ± 0.26, 1.35 ± 0.27, 1.27 ± 0.31, 1.23 ± 0.28, 1.20 ± 0.31 and 3.35 ± 0.78, 3.43 ± 0.84, 3.27 ± 0.71, 2.82 ± 0.66, 3.45 ± 0.77, 3.35 ± 0.79, respectively (Table 1). The mean ± SD ALT, AST, ALP, and HbA1c levels as well as the median (IQR) cholesterol, triglyceride, LDL-C, HDL-C, ALT, AST, ALP, and HbA1c levels for all six data categories are tabulated in Table 1. 

### 3.2. Comparative Status of Lipid Profile among All the Participants (n = 510)

**Figure 2:**
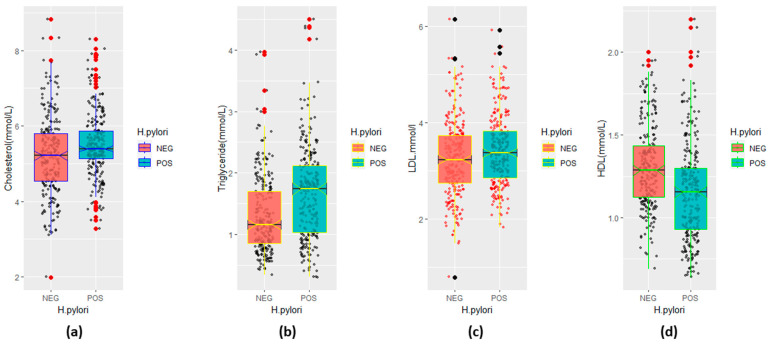
Illustration of lipid profile by *H. pylori*-infection status: (**a**) cholesterol disaggregated by infection status, (**b**) level of triglycerides by *H. pylori*-infection status, (**c**) LDL cholesterol by infection status, and (**d**) HDL cholesterol by status of *H. pylori* infection.

**Figure 3:**
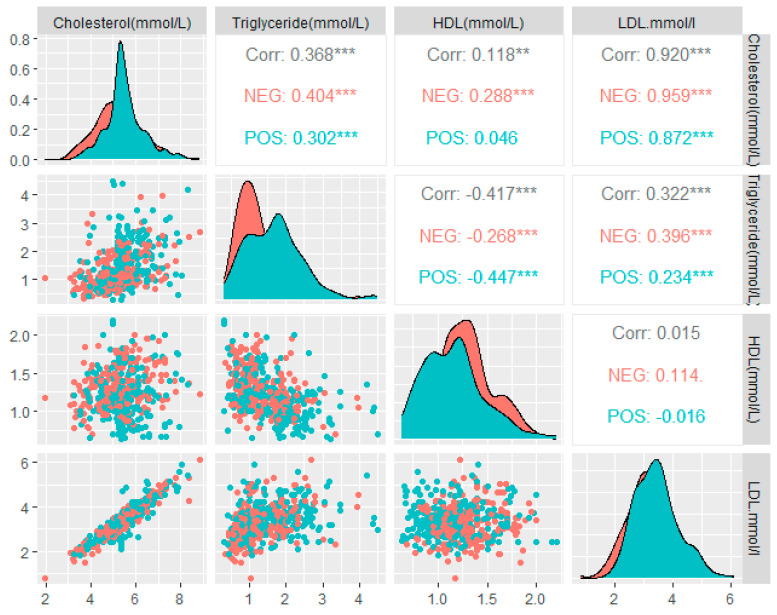
Correlogram of lipid profile by *H. pylori*-infection status: correlation coefficients (r) depicted in grey, red, and cyan colors represent the correlation between the parameter of the lipid profile in the overall, *H. pylori*−negative and *H. pylori*−positive individuals, respectively. Then number of asterisk defines the level of statistical significance.

The mean ± SD cholesterol, triglyceride, LDL-C, and HDL-C levels of the two groups (*H. pylori*-positive and *H. pylori*-negative participants) were compared to assess statistically significant differences by the *H. pylori*-infection status for all the six data categories. The levels of cholesterol (mean ± SD; 5.49 ± 0.85, 95%CI; 0.10–0.44), triglycerides (mean ± SD; 1.66 ± 0.75, 95%CI; 0.24–0.48), LDL-C (mean ± SD; 3.43 ± 0.74, 95%CI; 0.02–0.30), and HDL-C (mean ± SD; 1.15 ± 0.30, 95%CI; 0.10– 0.20) were measured in the *H. pylori*-positive participants for the overall categories. On the other hand, the levels of cholesterol (mean ± SD; 5.22 ± 1.0, 95%CI; 0.10–0.44), triglycerides (mean ± SD; 1.29 ± 0.62, 95%CI; 0.24–0.48), LDL-C (mean ± SD; 3.26 ± 0.81, 95%CI; 0.02–0.30), and HDL-C (mean ± SD; 1.30 ± 0.25, 95%CI; 0.10–0.20) were measured in the *H. pylori*-negative participants for the overall categories (Table 2). A statistically significant difference in the cholesterol (*p* < 0.01), triglyceride (*p* < 0.001), LDL-C (*p* < 0.05), and HDL-C (*p* < 0.001) levels was observed for the overall study participants. The differences in the mean ± SD LDL-C of the *H. pylori*-positive and *H. pylori*-negative participants (*p* > 0.05) in the male, female, age = 30–60, and age > 60 categories were not statistically significant. The differences in the mean ± SD cholesterol levels of the *H. pylori*-positive and *H. pylori*-negative participants (*p* > 0.05) in the female, age = 30–60, and age > 60 categories were not statistically significant. A significant difference was observed for the HDL-C levels (*p* < 0.001) across all the data categories. The comparative mean ± SD and *p*-values for all the four components of the lipid profile in the *H. pylori*-positive and -negative participants are summarized in detail in Table 2. The mean ± SD and 95%CI for each laboratory parameter of the lipid profile by *H. pylori* infection of all the six data categories are tabulated (Table 2). The median (IQR) cholesterol levels (Figure 2a), triglyceride levels (Figure 2b), LDL-C levels (Figure 2c), and HDL-C levels (Figure 2d) by *H. pylori*-infection status are illustrated in Figure 2. The correlation matrix between each component of the lipid profile and its distribution by *H. pylori*-infection status are depicted in Figure 3. A strong positive correlation was measured between the cholesterol and LDL levels in both the *H. pylori*-infected (r = 0.872, *p* < 0.001) and -uninfected participants (r = 0.959, *p* < 0.001) (Figure 3). A weak negative correlation was observed between the triglyceride and HDL levels in the *H. pylori*-positive participants (r = −0.447, *p* < 0.001). A comparatively weak negative correlation (r = −0.016, *p* > 0.05) was recorded between the HDL and LDL levels in the *H. pylori*-positive case (Figure 3).

### 3.3. The Magnitude of Imbalance in Lipid Profile among n = 510 Study Participants

By applying a two-sample test, the equality of proportion and the difference in the proportions of the infected individuals with altered lipid profiles and non-infected individuals with disturbed serum lipid profiles were calculated (Table 3). A statistically significant difference (*p* < 0.001) was observed among the overall proportion of hypercholesterolemia in the *H. pylori*-infected (N/%; 190/59.9%) and *H. pylori*-uninfected (N/%; 127/40%) participants (Table 3). A statistically significant difference (*p* < 0.001) was observed among the overall proportion of triglyceridemia in the *H. pylori*-infected (N/%; 136/68%) and *H. pylori*-uninfected (N/%; 64/32%) participants. The overall proportion of increased LDL-C and decreased HDL-C levels in the *H. pylori*-infected participants were (N/%; 234/53%) and (N/%; 149/71%), respectively (Table 3). On the other hand, the overall proportion of increased LDL-C and decreased HDL-C levels in the *H. pylori*-uninfected participants were (N/%; 201/46%) and (N/%; 60/28.7%), accordingly. A statistically significant difference (*p* < 0.01) was noticed in the proportions of an increased LDL-C as well as a decreased HDL-C of the *H. pylori*-infected and *H. pylori*-uninfected participants in the overall category of data (Table 3). The proportion (N/%) and *p*-value of all four components of dyslipidemia in the *H. pylori*-positive and -negative participants for the male, female, age < 30, age = 30–60, and age > 60 categories are summarized in detail in Table 3. The magnitude (number/proportion) of imbalance in the lipid profile (hypercholesterolemia, triglyceridemia, increased LDL levels, and decreased HDL levels) of males and females and ages < 30, ages > 30, age = 30–60, and ages > 60 are summarized in Table 3. The level of statistically significant differences (*p*-value) of the imbalance in lipid profiles in the *H. pylori*-positive and *H. pylori*-negative participants are also tabulated in Table 3 for the male, female, age < 30, age > 30, age = 30–60, and age > 60 categories. 

### 3.4. H. pylori as an Associated Factor of Dyslipidemia among All (n = 510) Study Participants

*H. pylori* as an associated factor of dyslipidemia (hypercholesterolemia, hypertriglyceridemia, increased LDL-C levels, and decreased HDL-C levels) was assessed by measuring the crude odds ratio (COR) and adjusted odds ratio (AOR) by applying univariate and bivariate logistic regression statistical models. The *p*-value and 95% confidence interval (95%CI) were also evaluated for each odds ratio (Table 4). The COR and AOR for age, gender, cholesterol levels, triglyceride levels, LDL-C levels, and HDL-C levels were also obtained by univariate and multivariate logistic regressions to gain insight into *H. pylori* as the associated factor of imbalance in lipid profiles (Table 5). The odds of having hypercholesterolemia (AOR: 2.64, 95%CI: 1.824–3.848, *p* < 0.001), hypertriglyceridemia (AOR: 3.24, 95%CI: 2.227–4.757, *p* < 0.001), increased LDL-C levels (AOR: 2.174, 95%CI: 1.309–3.684, *p* < 0.01), and decreased HDL-C levels (AOR: 4.2, 95%CI: 2.937–6.321, *p* < 0.001) are 2.64, 3.24, 2.17, and 4. 2 times higher in *H. pylori*-infected individuals as compared with the *H. pylori*-uninfected individuals (Table 4). For a one-unit increase in cholesterol, the odds of being infected by *H. pylori* increases by 226.2% (AOR: 3.26, 95%CI: 1.778–6.258, *p* < 0.001) (Table 5). For a one-unit increase in the level of triglycerides, the odds of being infected by *H. pylori* increase by a factor of 1.3 (AOR: 1.298, 95%CI: 0.928–1.834, *p* > 0.05). For an increase of one unit in the concentration of HDL-C, the odds of being infected by *H. pylori* decrease by a factor of 89% (AOR: 0.11, 95%CI: 0.043–0.267, *p* < 0.11). Meanwhile, with a one-unit increase in the concentration of LDL-C, the odds of being infected by *H. pylori* decrease by 67% (AOR: 0.333, 95%CI: 0.156–0.676, *p* < 0.01) (Table 5). Though the AORs for the age and gender of the study participants were not statistically significant (*p* > 0.05), the males’ crude odds of being *H. pylori*-infected was 1.6 times higher in comparison to the female participants (COR: 1.581, 95%CI: 1.116–2.246, *p* < 0.05).

## 4. Discussion

This study was designed to gain an insight into the association of the *H. pylori* infection with an imbalance in any component of the lipid profile (dyslipidemia), such as cholesterol levels, triglyceride levels, LDL-C levels, and HDL-C levels among *n* = 510 study individuals. The number and proportion of hypercholesterolemia, triglyceridemia, increased LDL-C levels, and decreased HDL-C levels in *H. pylori*-infected individuals was measured to be 68% (N; 136), 59.9% (N; 190), 53% (N; 234), and 71% (N; 149), which is tabulated in Table 4. Our findings are corroborated with that of various other studies [15,21,25]. Moreover, a statistically significant difference (*p* < 0.001) was observed in the overall proportion of hypertriglyceridemia and hypercholesterolemia between the *H. pylori*-infected and -uninfected individuals. A statistically significant difference (*p* < 0.01) was noticed in the proportions of an increased LDL-C as well as a decreased HDL-C of the *H. pylori*-infected and *H. pylori*-uninfected participants in the overall category of the data (Table 3). A statistically significant association of dyslipidaemia with the *H. pylori* infection has been reported in various other scientific studies [38,39,40,41].

*H. pylori* as an associated factor of dyslipidaemia was assessed in terms of the crude odds ratio (COR) and adjusted odds ratio (AOR) by applying a logistic regression statistical model (Table 4). Our results demonstrate that the odds of having hypercholesterolemia (AOR: 2.64, 95%CI: 1.824–3.848, *p* < 0.001), hypertriglyceridemia (AOR: 3.24, 95%CI: 2.227–4.757, *p* < 0.001), increased LDL-C levels (AOR: 2.174, 95%CI: 1.309–3.684, *p* < 0.01), and decreased HDL-C levels (AOR: 4.2, 95%CI: 2.937–6.321, *p* < 0.001) are 2.64, 3.24, 2.17, and 4.2 times higher in *H. pylori*-infected individuals compared with *H. pylori*-uninfected individuals (Table 4). Shimamoto et al. described clinically and statistically significant positive associations of the infection with cholesterol levels (standardized mean difference/SDM; 0.09, 95%CI; 0.07–0.10), triglyceride levels (SDM; 0.06, 95%CI; 0.05–0.08), and LDL-C levels (SDM; 0.11, 95%CI; 0.09–0.12), while a negative association was observed with HDL-C levels (SDM; −0.13, 95%CI; −0.14 to −0.12) in a meta-analysis study which corroborates our finding [42]. The dyslipidemia determined in the present study is also consistent with the findings of Adachi et al., who demonstrated a lower concentration of cholesterol, triglycerides, and LDL-C and a higher level of HDL-C in successfully eradicated *H. pylori* study subjects as compared to the subjects with persistent *H. pylori* infections [43]. 

Our results of hypercholesterolemia were found to be in line with the results of Tali et al., who reported a statistically significant association of an enhanced total cholesterol (OR: 2.3324, *p* = 0.0002) with the *H. pylori* infection [39]. Tali et al. also reported a statistically significant association of increased LDL-C levels (OR: 2.3, *p* = 0.0006) with the *H. pylori* infection, which corroborates the findings of our study [39]. *H. pylori*-associated increased LDL-C levels were reported by Takashima et al. [44]. Takashima et al. also described the decreased HDL-C levels in *H. pylori*-infected patients [44]. A significant association of the *H. pylori* infection with enhanced LDL-C levels and decreased levels of HDL-C was reported by Kanbay et al., which is consistent with our findings [45]. In addition to this, in a study on the association of serologically positive *H. pylori* infections with alterations in the lipid profile, considering a relatively larger sample number (*n* = 2573), accomplished by Haeri et al., decreased HDL-C levels and increased LDL-C levels were reported to be significantly (*p* < 0.03) associated with the *H. pylori* infection [46]. 

A significant association of increased LDL-cholesterolemia levels and decreased HDL-cholesterolemia levels with the *H. pylori* infection in a Japanese male population was described in a study accomplished by Satoh et al. [40]. These findings, in terms of high LDL-cholesterolemia levels and low HDL-cholesterolemia levels, are of paramount importance, as both are major risk factors for atherosclerosis and coronary diseases [47]. 

In our study, we found that with a one-unit increase in the level of cholesterol, triglycerides, and LDL-C, the odds of being infected by *H. pylori* increases by 226.2% (AOR: 3.26, 95%CI: 1.778–6.258, *p* < 0.001), 30% (AOR: 1.298, 95%CI: 0.928–1.834, *p* > 0.05), and 67% (AOR: 0.333, 95%CI: 0.156–0.676, *p* < 0.01), respectively (Table 5). On the other hand, for an increase of one unit in the concentration of HDL-C, the odds of being infected by *H. pylori* decreases by a factor of 89% (AOR: 0.11, 95%CI: 0.043–0.267, *p* < 0.001) (Table 5). Our results corroborate those of an investigation (a cross-sectional study, *n* = 37,263) on the association of *H. pylori* colonization and infection with lipid profiles accomplished by Kim et al., who reported the statistically significant impact of the *H. pylori* infection on hypercholesterolemia (*p*  <  0.001), hyper-LDL-cholesterolemia (*p*  <  0.001), and hypo-HDL-cholesterolemia [38]. Dyslipidemia is recognized as one of the major risk factors for cardiovascular diseases (CVDs). Our results show *H. pylori*-infection-dependent alterations in the lipid profile of *H. pylori*-infected individuals, which suggests an increased risk of CVDs in *H. pylori*-infected subjects, which is substantiated by the findings of Sun et al., who reported an association of an increased risk of composite CVDs and coronary heart disease (CHD) with the H. pylori infection [18]. Sun et al. reported a slight association of CVDs (risk ratio/RR: 1.10) and CHD (risk ratio/RR: 1.10) [18]. Jamkhande et al. explained the direct or indirect impact of the *H. pylori* infection in the development of CVDs [48]. Sharma et al. described the role of the Cag-A-seropositive *H. pylori* strain as a predisposing factor of CVDs [49]. Furthermore, Torres et al. also described the indirect role of the H. pylori infection in the development of atherothrombosis and CVDs [50]. Though the AOR for the age and gender categories of the study participants were not statistically significant (*p* > 0.05), the males’ crude odds of being *H. pylori*-infected was 1.6 times higher in comparison to the female participants (COR: 1.581, 95%CI: 1.116–2.246, *p* < 0.05). However, there are various other studies, in which authors have reported results that are contradictory to our results and which demonstrate that *H. pylori* infection does not impact the lipid profile or interfere with lipid metabolism. Regnstrom et al. measured a statistically non-significant difference between the lipid profile and *H. pylori* seropositivity. Additionally, a significant association between the lipid profile (cholesterol and triglyceride levels) and the *H. pylori* infection was not reported by Patel et al. [51]. Vafaeimanesh et al. also did not observe any significant association of the lipid profile with the *H. pylori* infection in their study population [52]. Such discrepant findings could be explained by differences in the methods of diagnosis of the *H. pylori* infection, sample size, and variation in the study population. 

The data for *H. pylori* detection was based only on rapid *H. pylori* antigen tests in the stool samples, which could be acknowledged as a limitation of the present study, and therefore, other methods of detection such as endoscopy, histopathological examinations of biopsies and the detection of genetic material in conjunction with serological tests (antigen and antibody) should be adopted to gain insights of the association between the *H. pylori* infection and dyslipidemia inquisitively. Although *H. pylori*-infected, severely ill patients who were kept on the long-term use of proton pump inhibitors (PPIs) were excluded from this study, the access to insufficient information on the short-term or intermittent use of PPIs by the study participants could be documented as a limitation of this study. 

## 5. Conclusions

Our results demonstrate the enhanced risk of dyslipidemia in *H. pylori*-infected individuals compared to *H. pylori*-negative individuals. An increased risk of dyslipidemia is the major risk factor for cardiovascular and cerebrovascular disorders. The occurrence of dyslipidemia in *H. pylori*-infected individuals may trigger atherosclerosis, coronary heart disease, and cerebral infarction in the population. However, some reports do not elucidate the enhanced risk of dyslipidemia in *H. pylori*-infected individuals and that variation could be population-dependent; therefore, it is recommended to undertake such studies in different populations to achieve a full insight into this. Given the statistically significant association of dyslipidemia with *H. pylori*-positive individuals, the effective management of the lipid profile of *H. pylori*-infected patients would be beneficial to minimize the clinical impact of the infection on cardiovascular diseases. It would be interesting to accomplish a temporal assessment of the serum lipid profile concerning infections in *H. pylori*-infected patients to gain a deeper insight into the impact of the *H. pylori* infection on dyslipidemia. 

## Figures and Tables

**Table 1:** Tabulation of baseline characteristics of all the participants (*n* = 510) stratified into six categories.

Variables	Statistics	Categories					
		Overall	Male	Female	Age < 30 (In Years)	Age = 30–60 (In Years)	Age > 60 (In Years)
Age (In years)	MEDIAN (IQR)	43.0 (53.0–35.0)	42.0 (52.0–33.0)	45.0 (54.0–36.0)	24.0 (27.0–19.0)	44.00 (51.00–37.00)	66.0 (70.00–63.0)
	Mean ± SD	44.01 ± 13.58	43.31 ± 13.81	44.69 ± 13.33	23.01 ± 5.45	44.01 ± 8.10	67.29 ± 5.83
Cholesterol (mmol/L)	MEDIAN (IQR)	5.34(5.83–4.75)	5.37 (5.83–4.85)	5.30 (5.84–4.71)	4.71 n(5.36–4.21)	5.39 (5.90–4.92)	5.43 (5.89–5.01)
	Mean ± SD	5.36 ± 0.96	5.38 ± 1.02	5.34 ± 0.92	4.78 ± 0.89	5.47 ± 0.93	5.38 ± 1.05
Triglyceride (mmol/L)	MEDIAN (IQR)	1.35(1.89–0.91)	1.60 (1.98– 1.04)	1.20 (1.79–0.86)	0.99 (1.73–0.70)	1.36 (1.91–0.94)	1.65 (1.92–1.08)
	Mean ± SD	1.49 ± 0.72	1.60 ± 0.74	1.37 ± 0.68	1.18 ± 0.65	1.52 ± 0.73	1.20 ± 0.66
HDL (mmol/L)	MEDIAN (IQR)	1.22 (1.39–1.02)	1.09 (1.26–0.93)	1.31 (1.50–1.16)	1.21 (1.49–1.07)	1.22 (1.39–1.04)	1.23 (1.37–0.95)
	Mean ± SD	1.23 ± 0.29	1.11 ± 0.26	1.35 ± 0.27	1.27 ± 0.31	1.23 ± 0.28	1.20 ± 0.31
LDL (mmol/L)	MEDIAN(IQR)	3.33 (3.76–2.78)	3.38 (3.90–2.87)	3.24 (3.74–2.74)	2.76 (3.16–2.27)	3.41 (3.89–2.92)	3.35 (3.76–2.79)
	Mean ± SD	3.35 ± 0.78	3.43 ± 0.84	3.27 ± 0.71	2.82 ± 0.66	3.45 ± 0.77	3.35 ± 0.79
ALT.U/L	MEDIAN (IQR)	20.0 (28.0–14.25)	25.0(35.0–20.0)	16.0 (21.0–12.0)	16.0 (27.75–11.00)	21.0 (30.0–16.0)	19.0 (22.0–15.0)
	Mean ± SD	24.16 ± 16.58	31.11 ± 19.99	17.38 ± 7.77	22.11± 20.49	25.21 ± 16.60	20.19 ± 9.51
AST.U/L	MEDIAN (IQR)	22.0 (27.0–18.0)	24.0 (29.0–22.0)	20.0 (24.0–16.25)	20.0 (27.0–17.25)	22.0 (27.0–18.0)	23.0 (25.50–19.0)
	Mean ± SD	23.9 ±8.68	26.66 ± 9.36	21.19 ± 6.97	22.53 ± 7.41	24.29 ± 9.13	23.06 ± 6.90
ALP.UL	MEDIAN (IQR)	68.0 (82.75–57.0)	70.0 (87.0–60.75)	66.0 (81.0–53.0)	74.0 (94.25–59.50)	67.00 (81.0–57.0)	68.0 (80.50–58.50)
	Mean ± SD	74.84 ± 35.07	78.44± 35.66	71.32 ± 34.20	91.79 ± 63.52	71.49 ± 21.15	76.02 ± 50.02
HbA1c (%)	MEDIAN(IQR)	5.5(5.8–5.1)	5.5 (5.8–5.1)	5.5 (5.88–5.2)	5.20 (5.48–5.0)	5.5 (5.8–5.2)	6.00 (6.70–5.50)
	Mean ± SD	5.77 ± 2.94	5.87 ± 4.09	5.68 ± 0.86	5.24 ± 0.28	5.76 ± 3.36	6.44 ± 1.32

ALP = Alkaline phosphatase, SD = standard deviation, Hb = hemoglobin, IQR = interquartile range, AST = aspartate aminotransferase, ALT = alanine aminotransferase.

**Table 2:** Comparative status of lipid profile by *Helicobacter pylori*-infection status (by applying Welch’s two sample *t*-test and the SD function).

Overall Participants (*n* = 510)			
Lipid Profile	*Helicobacter pylori*-Infection Status		*p*-Value
	*H. pylori* Negative (*n* = 250)	*H. pylori* Positive (*n* = 260)	
	Mean ± SD	Mean ± SD	
Cholesterol (mmol/L)	5.22 ± 1.0	5.49 ± 0.85	*p* < 0.01
Triglyceride (mmol/L)	1.29 ± 0.62	1.66 ± 0.75	*p* < 0.001
LDL-C (mmol/L)	3.26 ± 0.81	3.43 ± 0.74	*p* < 0.05
HDL-C (mmol/L)	1.30 ± 0.25	1.15 ± 0.30	*p* < 0.001
Male participants (*n* = 252)			
	*H. pylori* negative (*n* = 109)	*H. pylori* positive (*n* = 143)	*p*-value
Cholesterol (mmol/L)	5.19 ± 1.02	5.53 ± 0.85	*p* < 0.05
Triglycerides (mmol/L)	1.43 ± 0.67	1.73 ± 0.75	*p* < 0.01
LDL-C (mmol/L)	3.32 ± 0.90	3.51 ± 0.78	*p* > 0.05
HDL-C (mmol/L)	1.18 ± 0.21	1.05 ± 0.28	*p* < 0.001
Female participants (*n* = 258)			
	*H. pylori* negative (*n* = 141)	*H. pylori* positive (*n* = 117)	*p*-value
Cholesterol (mmol/L)	5.24 ± 0.94	5.45 ± 0.86	*p* > 0.05
Triglycerides (mmol/L)	1.19 ± 0.57	1.58 ± 0.74	*p* < 0.001
LDL-C (mmol/L)	3.21 ± 0.73	3.33 ± 0.67	*p* > 0.05
HDL-C (mmol/L)	1.40 ± 0.24	1.27 ± 0.27	*p* < 0.001
Age category (Age < 30, *n* = 70)			
	*H. pylori* negative (*n* = 40)	*H. pylori* positive (*n* = 30)	*p*-value
Cholesterol (mmol/L)	4.49 ± 0.91	5.16 ± 0.69	*p* < 0.001
Triglyceride (mmol/L)	0.88 ± 0.42	1.56 ± 0.70	*p* < 0.001
LDL-C (mmol/L)	2.68 ± 0.67	3.00 ± 0.58	*p* < 0.05
HDL-C (mmol/L)	1.35 ± 0.26	1.15 ± 0.33	*p* < 0.01
Age category (Age = 30–60, *n* = 377)			
	*H. pylori* negative (*n* = 180)	*H. pylori* positive (*n* = 197)	*p*-value
Cholesterol (mmol/L)	5.39 ± 1.0	5.53 ± 0.83	*p* > 0.05
Triglycerides (mmol/L)	1.36 ± 0.63	1.66 ± 0.78	*p* < 0.001
LDL-C (mmol/L)	3.40 ± 0.79	3.48 ± 0.74	*p* > 0.05
HDL-C (mmol/L)	1.29 ± 0.24	1.17 ± 0.29	*p* < 0.001
Age category (Age > 60, *n* = 63)			
	*H. pylori* negative (*n* = 30)	*H. pylori* positive (*n* = 33)	*p*-value
Cholesterol (mmol/L)	5.17 ± 0.99	5.57 ± 1.01	*p* > 0.05
Triglycerides (mmol/L)	1.46 ± 0.64	1.75 ± 0.64	*p* > 0.05
LDL-C (mmol/L)	3.17 ± 0.77	3.50 ± 0.79	*p* > 0.05
HDL-C (mmol/L)	1.33 ± 0.279	1.06 ± 0.28	*p* < 0.001

**Table 3:** The magnitude of dyslipidemia among the *H. pylori*-infected and -uninfected study participants in the Asir region of Saudi Arabia (2-sample test for equality of proportions and Pearson’s Chi-squared test to assess *p*-values).

Overall Participants			
Dyslipidemia	*Helicobacter pylori*-Infection Status		*p*-Value
	*H. pylori* Negative	*H. pylori* Positive	
	N (%)	N (%)	
Hypercholesterolemia	127 (40%)	190 (59.9%)	*p* < 0.001
Hypertriglyceridemia	64 (32%)	136 (68%)	*p* < 0.001
Increased LDL-C	201 (46%)	234 (53%)	*p* < 0.01
Decreased HDL-C	60 (28.7%)	149 (71%)	*p* < 0.001
Male participants			
Hypercholesterolemia	56 (33.5%)	111 (66.4%)	*p* < 0.001
Hypertriglyceridemia	36 (30.7%)	81 (69.2%)	*p* < 0.001
Increased LDL-C	88 (40%)	131 (59.8%)	*p* < 0.05
Decreased HDL-C	14 (16%)	73 (83.9%)	*p* < 0.001
Female participants			
Hypercholesterolemia	71 (47.3%)	79 (52.6%)	*p* < 0.01
Hypertriglyceridemia	28 (33.7%)	55 (66.2%)	*p* < 0.001
Increased LDL-C	113 (52.3%)	103 (47.6%)	*p* > 0.05
Decreased HDL-C	46 (37.7%)	76 (62.2%)	*p* < 0.001
Age category (Age < 30)			
Hypercholesterolemia	7 (25%)	21 (75%)	*p* < 0.001
Hypertriglyceridemia	3 (10.5%)	16 (84.2%)	*p* < 0.001
Increased LDL-C	22 (48%)	23 (51.1%)	*p* > 0.05
Decreased HDL-C	8 (36.3%)	14 (63.6%)	*p* < 0.05
Age category (Age = 30–60)			
Hypercholesterolemia	104 (42.1%)	143 (57.8%)	*p* < 0.01
Hypertriglyceridemia	50 (33.1%)	101 (66.8%)	*p* < 0.001
Increased LDL-C	156 (46.1%)	182 (53.8%)	*p* > 0.05
Decreased HDL-C	44 (28.3%)	111 (71.6%)	*p* < 0.001
Age category (Age > 60)			
Hypercholesterolemia	16 (38%)	26 (61.9%)	*p* > 0.05
Hypertriglyceridemia	11 (36.6%)	19 (63.3%)	*p* > 0.05
Increased LDL-C	23 (44.2%)	29 (55.7%)	*p* > 0.05
Decreased HDL-C	8 (25%)	24 (75%)	*p* < 0.001

**Table 4:** Univariate and bivariate logistic regression analyses for assessing *H. pylori*-associated dyslipidemia.

Dyslipidemia by *H. pylori* Infection	Univariate BLR		Bivariate BLR	
	Unadjusted OR/COR		Adjusted OR (AOR)	
	COR	*p*-Value	AOR	*p*-Value
Hypercholesterolemia	2.629	*p* < 0.001	2.641	*p* < 0.001
Triglyceridemia	3.187	*p* < 0.001	3.241	*p* < 0.001
Increased LDL-C	2.194	*p* < 0.01	2.174	*p* < 0.01
Decreased HDL-C	4.251	*p* < 0.001	4.287	*p* < 0.001

BLR = binary logistic regression; COR = crude odds ratio; OR = odds ratio; AOR = adjusted odds ratio, adjusted for age and HbA1c of all the participants; reference/dummy category = *H. pylori*-negative participants.

**Table 5:** Univariate and multivariate logistic regression analyses for determining the association of *H. pylori* infection with age, gender, and all four parameters of the lipid profile.

Demographic and Lipid Profile by *H. pylori* Infection	Univariate BLR		Multivariate BLR	
	Unadjusted OR/COR		Adjusted OR (AOR)	
	COR	*p*-Value	AOR	*p*-Value
Age (in years)	1.003	*p* > 0.05	0.994	*p* > 0.05
Gender	1.581	*p* < 0.05	1.060	*p* > 0.05
Cholesterol (mmol/L)	1.357	*p* < 0.01	3.262	*p* < 0.001
Triglyceride (mmol/L)	2.195	*p* < 0.001	1.298	*p* > 0.05
LDL-C (mmol/L)	1.314	*p* < 0.05	0.333	*p* < 0.01
HDL-C (mmol/L)	0.143	*p* < 0.001	0.110	*p* < 0.001

BLR = binary logistic regression; COR = crude odds ratio; OR = odds ratio; AOR = adjusted odds ratio, adjusted for all 5 parameters except for the ones for which the odds ratio has been described in the table. The reference category for gender was female. The reference category for the outcome variable was *H. pylori*-uninfected individuals.

## Data Availability

The data supporting these research findings are not publicly available to protect the privacy of the research participants. However, they are available from the corresponding author.
